# The Role of Branched-Chain Amino Acids in Nutrient Allocation of the Porcine Placenta: A Review

**DOI:** 10.3390/ani16182851

**Published:** 2026-09-10

**Authors:** Jun Bai, Yiru Wang, Xinhang Li, Jiajun Li, Zhenlong Wu, Xinjian Li, Kejun Wang, Ruimin Qiao, Tong Fu, Yanpeng Li

**Affiliations:** 1 College of Animal Science and Technology, Henan Agricultural University, Zhengzhou 450046, China; baijun0320@163.com (J.B.); w13663726642@163.com (Y.W.); 19882957970@163.com (X.L.); j2529119937@sina.com (J.L.); wangkejun.me@163.com (K.W.); qrm480@163.com (R.Q.); 2 State Key Laboratory of Animal Nutrition, Department of Companion Animal Science, College of Animal Science and Technology, China Agricultural University, Beijing 100193, China; wuzhenlong@cau.edu.cn; 3 Sanya Institute, Hainan Academy of Agricultural Sciences, Sanya 571100, China; lxjlongfei@163.com; 4 Muyuan United Industrial Research Institute, Henan Agricultural University, Zhengzhou 450046, China; 5 Muyuan Foods Group Co., Ltd., Nanyang 473000, China

**Keywords:** branched-chain amino acids, placenta, nutrient allocation, mTOR signaling, amino acid transporters

## Abstract

During swine gestation, the placenta serves as the sole interface for nutrient, gas, and signaling molecule exchange between the dam and fetuses, and its function critically determines pregnancy outcomes. Branched-chain amino acids (BCAAs)—leucine, isoleucine, and valine—are substrates for protein synthesis, precursors for glutamate and glutathione, and signaling molecules that activate the mechanistic target of rapamycin (mTOR) pathway. This review synthesizes current evidence on BCAA transport, catabolism, and signaling in the porcine placenta, organized around four mechanistic axes: transporter-mediated transplacental transfer, local catabolism supporting energy production and antioxidant defense, mTORC1-dependent regulation of growth and differentiation, and modulation of endocrine and immune homeostasis. Both deficiency and excess of BCAAs compromise placental function, contributing to intrauterine growth restriction and metabolic programming abnormalities through a U-shaped dose–response relationship. Critical knowledge gaps remain, including the reliance on rodent and in vitro models, the absence of standardized supplementation protocols and dose–response data, unaddressed confounders (parity, litter size, environment), and the lack of formal power analyses or meta-analytic synthesis. We propose precision dietary strategies to optimize BCAA supplementation during gestation for improved reproductive performance in swine.

## Simple Summary

Piglet survival and birth weight are critical to the swine industry, and the placenta is the organ that determines how much nutrition each developing piglet receives. Yet we still know relatively little about which specific nutrients control placental function. This review looks at how three essential nutrients called branched-chain amino acids—leucine, isoleucine, and valine, which pigs must obtain from their diet—affect placental development in pregnant sows. What makes these amino acids interesting is that they do not just serve as raw materials for building proteins; they also switch on a key growth pathway inside placental cells. We found that they support placental function in four main ways: crossing from mother to fetus to nourish the developing piglet, providing energy to placental cells and protecting them from damage, triggering new blood vessel formation and cell growth, and helping maintain hormonal and immune balance. Importantly, both too little and too much of these amino acids can damage the placenta and restrict fetal growth. This means the amount and the balance among the three need to be carefully adjusted at different stages of pregnancy. Doing so could help farmers improve piglet survival, increase birth weight, and reduce economic losses in pig production.

## 1. Introduction

Mother nutrient status during gestation affects fetal development and birth outcomes for swine production. The placenta is the major connection between mother and fetus, which determines nutrient, gas, and signaling molecule exchange, and thus fetal growth potential and survival. Placental efficiency in sows determines litter size as well as piglet birth weight and uniformity, which are both economic aspects of swine industries [1]. Branched-chain amino acids (BCAAs)—leucine, isoleucine, and valine—are not only fundamental substrates for protein synthesis but also act as critical signaling molecules modulating cellular proliferation, metabolism, and autophagy through pathways such as mTOR and AMPK [2]. Recent studies show that BCAAs regulate placental shape, vascularization, expression of transporter genes, and energy metabolism. Dietary leucine supplementation at late gestation increased newborn piglet birth weight by stimulating placental protein synthesis and catalyzing glycolytic and fatty acid oxidation through PI3K/Akt/mTOR activation [3]. Placental vascular development and angiogenesis are important for fetal growth. It can be affected by BCAA availability, because placental insufficiency with impaired villus development and reduced vascular density can be a main factor for intrauterine growth restriction (IUGR) in swine. Also, BCAAs can interact with glucose and fatty acid metabolic pathways through the signaling cascades related to the mTORC1 hub [4]. Environmental and pathological factors, including maternal obesity and cadmium exposure, as well as inflammatory conditions, can affect placental function and fetal outcomes, which makes BCAA-mediated nutrient allocation complex [5,6]. Here, we examine the transport, metabolism, signaling, and function of BCAAs in porcine placental nutrient allocation and their potential as nutritional intervention targets for improving reproduction efficiency in sows. While amino acid nutrition in swine reproduction has been broadly studied, BCAAs merit separate consideration for several reasons: (1) they serve as direct allosteric activators of mTORC1 via the Sestrin2–GATOR2 axis, distinguishing them from other amino acids that act primarily as substrates; (2) the ‘leucine paradox’—whereby excess leucine depletes all three BCAAs through competitive catabolism—creates unique dose–response complexities; and (3) sow-specific data on BCAA placental transport and metabolism are less extensively characterized mechanistically compared to the extensive human and rodent literature. This review addresses these gaps by systematically evaluating BCAA-mediated placental regulation in swine, integrating mechanistic evidence from porcine, murine, and human studies where appropriate. Spatial transcriptomics and single-cell RNA sequencing have not yet been widely applied to the porcine placenta but hold potential for mapping cell type-specific BCAA responses. It is important to note that this review integrates evidence from porcine, murine, and human studies. While this comparative approach broadens the mechanistic framework, species differences in placental structure must be acknowledged: the porcine placenta is epitheliochorial (six tissue layers), whereas murine and human placentae are hemochorial (three layers with direct maternal blood contact). These structural differences fundamentally alter nutrient transport mechanisms and the translational applicability of cross-species findings. Where murine or human data are cited, we note the species of origin and discuss the biological plausibility of translational relevance.

### Literature Search Strategy

This narrative review was constructed by searching PubMed, Web of Science, and Scopus for articles published between 2010 and 2025 using the terms ‘branched-chain amino acids’ AND (‘placenta’ OR ‘pregnancy’ OR ‘gestation’) AND (‘swine’ OR ‘porcine’ OR ‘sow’). Additional records were identified from reference lists of retrieved articles. We included original research articles, systematic reviews, and meta-analyses addressing BCAA transport, metabolism, signaling, or supplementation in the context of placental function. Studies in rodent and human models were included where they provided mechanistic insights relevant to porcine physiology, with species origin explicitly noted throughout the text.

## 2. Materials and Methods

This review was conducted in the form of a narrative synthesis, aiming to integrate the current evidence on the roles of branched-chain amino acids (BCAAs)—leucine, isoleucine, and valine—in placental nutrient allocation, metabolic regulation, and signaling during sow gestation, with particular attention to transplacental BCAA transport mechanisms, the mTOR signaling pathway, angiogenesis, endocrine, and immune regulatory functions, as well as the effects of BCAA deficiency or excess on placental function and fetal development. Although structurally narrative in nature, systematic elements were incorporated to enhance transparency and reproducibility. A structured literature search was conducted using the electronic databases PubMed, Web of Science, and Scopus. The search encompassed records published through June 2026. The search strategy employed combinations of the following keywords: branched-chain amino acids, BCAAs, leucine, isoleucine, valine, placenta, pregnancy, gestation, swine, porcine, sow, mTOR signaling, and angiogenesis.

Studies were considered eligible for inclusion if they met at least one of the following criteria: described the transport, metabolism, or signaling functions of branched-chain amino acids (leucine, isoleucine, and/or valine) in the porcine placenta or related trophoblast models; investigated the effects of BCAA supplementation or deficiency on placental development, angiogenesis, endocrine function, immune regulation, or fetal growth in swine; employed in vitro, in vivo, or ex vivo methodological approaches to examine BCAA-mediated pathways (e.g., mTOR, PI3K/Akt, HIF-1α) in placental or trophoblast systems; or elucidated the associations between BCAAs and porcine reproductive physiology, fertility, or placental disorders. Priority was given to studies with clearly described experimental designs, standardized sample collection procedures, and transparent analytical workflows, particularly those involving metabolomic and gene expression analyses. Non-porcine studies were included only when they provided mechanistic or comparative insights directly relevant to porcine placental biology, with the species of origin explicitly indicated in the interpretation of results.

Publications were excluded if they did not directly address the interactions between BCAAs and placental function, lacked sufficiently detailed methodological information, or consisted entirely of conference abstracts, non-peer-reviewed sources, or duplicate reports. No strict language restrictions were imposed; however, English-language peer-reviewed publications were preferentially considered. Titles and abstracts were screened for relevance, followed by full-text evaluation of eligible studies. The reference lists of all included articles were manually screened to identify additional relevant publications.

Although a formal risk of bias scoring system was not applied, all included studies were critically appraised for methodological rigor, including the appropriateness of experimental design, clarity of BCAA supplementation protocols (dose, duration, and gestational stage), validity of analytical methods (amino acid quantification, gene/protein expression, and metabolomic profiling), and transparency of reporting. This qualitative assessment ensured that methodological limitations were explicitly considered when interpreting the synthesized findings. Due to substantial heterogeneity across studies in experimental designs, BCAA supplementation protocols, analytical platforms, and reporting standards, a formal quantitative meta-analysis was not feasible. Findings were, therefore, synthesized using a qualitative, thematic approach, with evidence organized according to mechanistic pathway, BCAA type, and clinical relevance.

This study did not involve animal experimentation or sample collection; therefore, ethical approval was not required.

## 3. Sow Placental Structure, BCAA Transport, Metabolism, and mTOR Signaling

### 3.1. Placental Structure and BCAA Transport

Porcine placenta is epitheliochorial. It is a complicated interface formed by interdigitation of the maternal uterine endometrium microfolds and of the fetal chorionic microvilli. The microanatomical structure provides a large surface area for mother–fetal exchange and carries gases, nutrients, and waste products required for development. Unlike hemochorial or endotheliochorial placentae, the epitheliochorial design retains all six tissue layers between the maternal and fetal blood supplies. They are essential for pigs as they compensate for the absence of direct contact between mother and fetus by maximizing the absorptive and secretory interface [7]. Placental development in pigs goes through several stages: first attachment, vascularization, and functional differentiation. The attachment stage establishes a smooth connection between the trophoblast and the uterine epithelium via extracellular vesicles (EVs) carrying miRNAs and proteins, signaling trophoblast growth, migration, and invasion, which are essential for implantation and placental formation. Then, angiogenesis starts, and the blood vessels begin to form. Maternal and fetal vasculature can be remodeled by angiogenic factors including VEGFA, NOS3, FGF9, and other related proteins, and this process is regulated by these factors so that blood vessel formation can be controlled in placental development. Also, functional differentiation continues, and the placental regions start to change. Specific placental regions can specialize in nutrient transport, gas exchange, hormone secretion, and other functions, allowing different placental regions to specialize in distinct functions that support fetal growth. Disruptions in any stage can affect placental efficiency and fetal viability. Gene dysregulation studies of allogeneic pig pregnancies can provide evidence [8]. The extensive folding of the epitheliochorial interface establishes the anatomical basis for efficient BCAA transport and nutrient delivery to the developing fetus.

**Figure 1:**
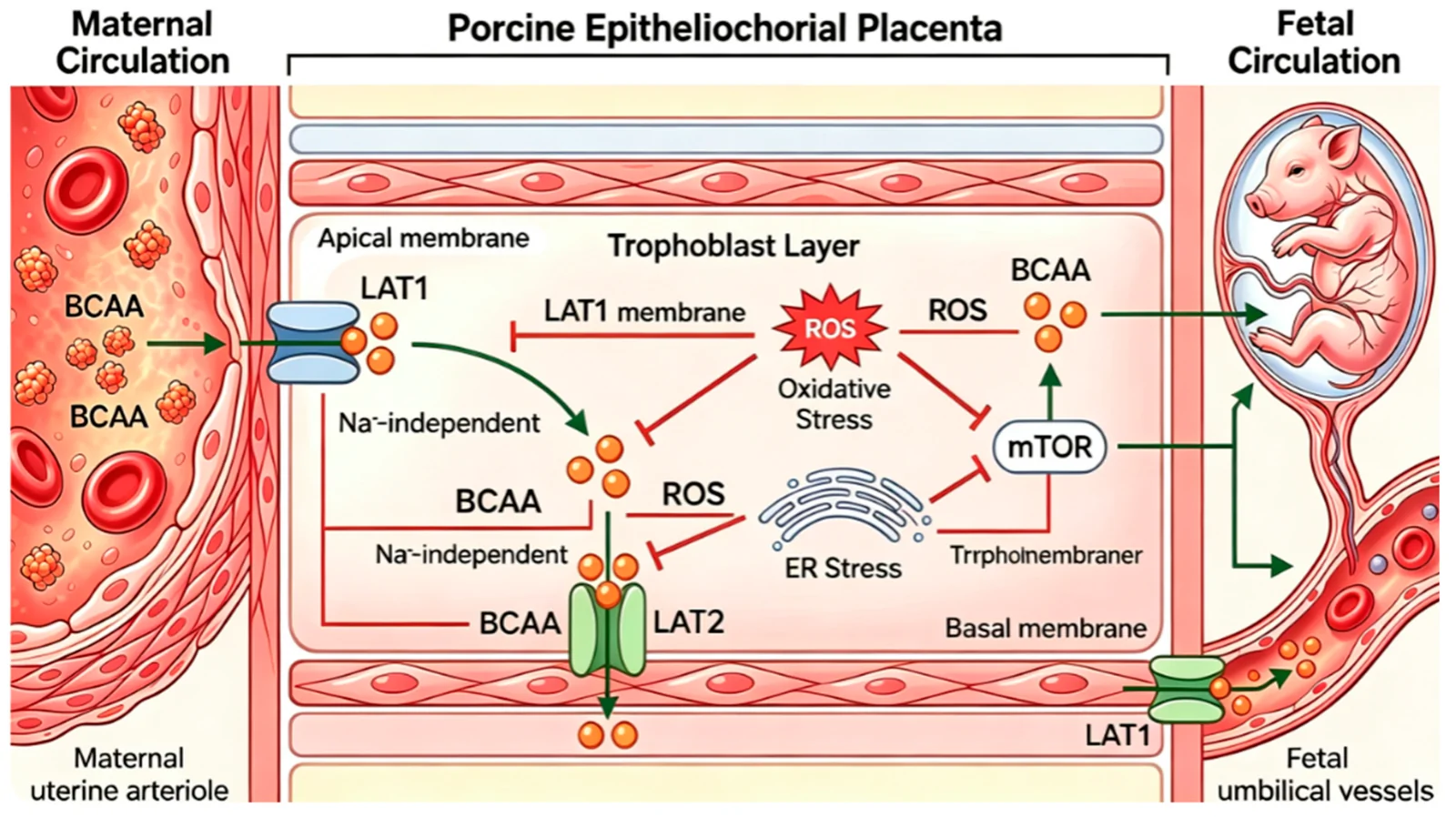
Branched-chain amino acids (BCAAs) transport mechanisms across the porcine epitheliochorial placenta. The porcine epitheliochorial placenta retains all six tissue layers between maternal and fetal circulation: (1) maternal uterine epithelium, (2) maternal connective tissue, (3) maternal capillary endothelium, (4) fetal trophoblast, (5) fetal connective tissue, and (6) fetal capillary endothelium. System L transporters—LAT1 (SLC7A5) and LAT2 (SLC7A8)—mediate Na^+^-independent BCAA exchange across the trophoblast. Based on human trophoblast studies, LAT1 is presumed to localize to the apical membrane and LAT2 to the basal membrane, though this polarization in the porcine placenta requires direct confirmation—mediate Na^+^-independent BCAA exchange across the trophoblast. Additional transporters including system B^0+^ (ATB^0+^) and system y+L (SLC7A6/7A7) contribute to directional BCAA transfer. BCAAs delivered to the fetal circulation support protein synthesis and fetal growth, while oxidative and ER stress in trophoblasts may modulate transport efficiency and mTOR signaling. Created in Biorender. Yiru Wang. (2026) https://BioRender.com.

The porcine epitheliochorial placenta, which is highly folded and coordinated at the heart of fetal development, establishes the basis for efficient BCAA transport and nutrient access to the developing fetus. BCAAs and amino acids transport essential nutrients, such as those from maternal circulation, to the developing fetus in the placental vasculature and are the backbone of its metabolism. BCAAs, such as leucine, isoleucine, and valine, are important for fetal growth and development as substrates for protein synthesis and signaling molecules that regulate metabolism. The trophoblast layer (the syncytiotrophoblast) expresses multiple amino acid transporters, with the system L transporters (LAT1/SLC7A5 and LAT2/SLC7A8) leading the Na^+^-independent exchange of large neutral amino acids (e.g., BCAA and aromatic amino acids) (Table 1). The system L transporter carries out an antiport mechanism and exchanges intracellular amino acids for extracellular substrates, enabling the accumulation of essential amino acids in the fetal compartment. Their activity is regulated by substrate availability and hormonal signals and adapts to changing maternal–fetal nutrient demands [9,10,11]. Moreover, the placenta’s capacity to transport amino acids is modulated by local metabolic imprinting and stress responses, including oxidative and endoplasmic reticulum stress, which can impair transporter function and contribute to pregnancy complications, such as fetal growth restriction [12]. In addition to system L, other transporter systems, such as system B^0+^ and system y+L, contribute to the transplacental transport of BCAAs, with differential localization within the polarized trophoblast layer establishing a directional gradient that facilitates efficient and selective amino acid transfer from mother to fetus [13,14]. In vitro studies using human trophoblast models have demonstrated that LAT1 mediates leucine uptake at the apical membrane and LAT2 facilitates efflux at the basal membrane; whether this polarized distribution is conserved in the porcine epitheliochorial placenta remains to be directly confirmed toward the fetal circulation, indicating a coordinated transcellular transport mechanism [15,16]. Expression and function can be regulated during trophoblast differentiation and syncytialization [17,18]. Beyond their role as transport substrates, BCAAs function as signaling molecules that regulate placental metabolism and transporter expression. BCAAs are activators of the mTOR signaling pathway in the trophoblast cells, modulating protein synthesis and cell growth and affecting the abundance of nutrient transporters [19]. When placental mTOR activity is increased, it is related to higher system A and system L transporter activity. This helps promote amino acid transfer and fetal growth. However, inhibition of mTOR signaling is associated with reduced transporter expression and fetal growth restriction [20]. Maternal nutritional deficiencies or metabolic disturbances can alter placental BCAA sensing and signaling, and this leads to maladaptive changes in transporter expression and nutrient flux [21,22]. All system L transporters (LAT1 and LAT2) and complementary system B^0^-1 and y-1L collectively lead to selective and efficient BCAA transfer between the placenta, and their expression and activity are the fastest way to achieve the increase in fetal BCAA demand. BCAA transport across the placenta is mediated by several factors. One of the most important factors is the concentration gradient of the amino acids in maternal blood. Maternal plasma BCAAs increase drive placental transport, and variations in maternal BCAA metabolism in pregnancy can alter the amino acid content of the placental microenvironment. In addition to substrate availability, the placental vascular network and blood flow drive BCAA delivery. The placental vascular network undergoes extensive remodeling and angiogenesis during pregnancy. Angiogenic factors such as VEGF control placental vasculature, and newly found evidence suggests that BCAAs would be able to control angiogenic signaling by mediators such as VEGF, providing feedback whereby BCAAs could regulate their own transport capability. Disruptions in angiogenic signaling (e.g., preeclampsia or maternal obesity) can degrade placental blood flow and reduce BCAA supply to the fetus [23,24]. BCAAs are also connected by competition with other amino acids. Amino acid transporters for various amino acids are both heterogeneous substrate types and compete between amino acids such as arginine, glutamine, or BCAAs. Hormonal and local signalling controls placental nutrient transport; insulin and IGF-1 regulate the expression and activity of a transporter, and endogenous inhibitors like DEPTOR regulate mTOR signalling and amino acid transport [25,26,27]. In conclusion, placental nutrient allocation is a process with strict control mechanisms that includes maternal nutrient supply, the transfer process mediated by selective transporters in the placental barrier, and finally, the utilization of these nutrients by the fetus. BCAAs can work as substrates and signaling molecules at the same time. They influence placental metabolism, and they can change transporter expression. Understanding these regulatory networks is essential for elucidating the pathophysiology of pregnancy complications and developing targeted interventions to optimize fetal nutrition (Figure 1).

### 3.2. BCAA Metabolism, mTOR Signaling, and Placental Vascular Development

**Figure 2:**
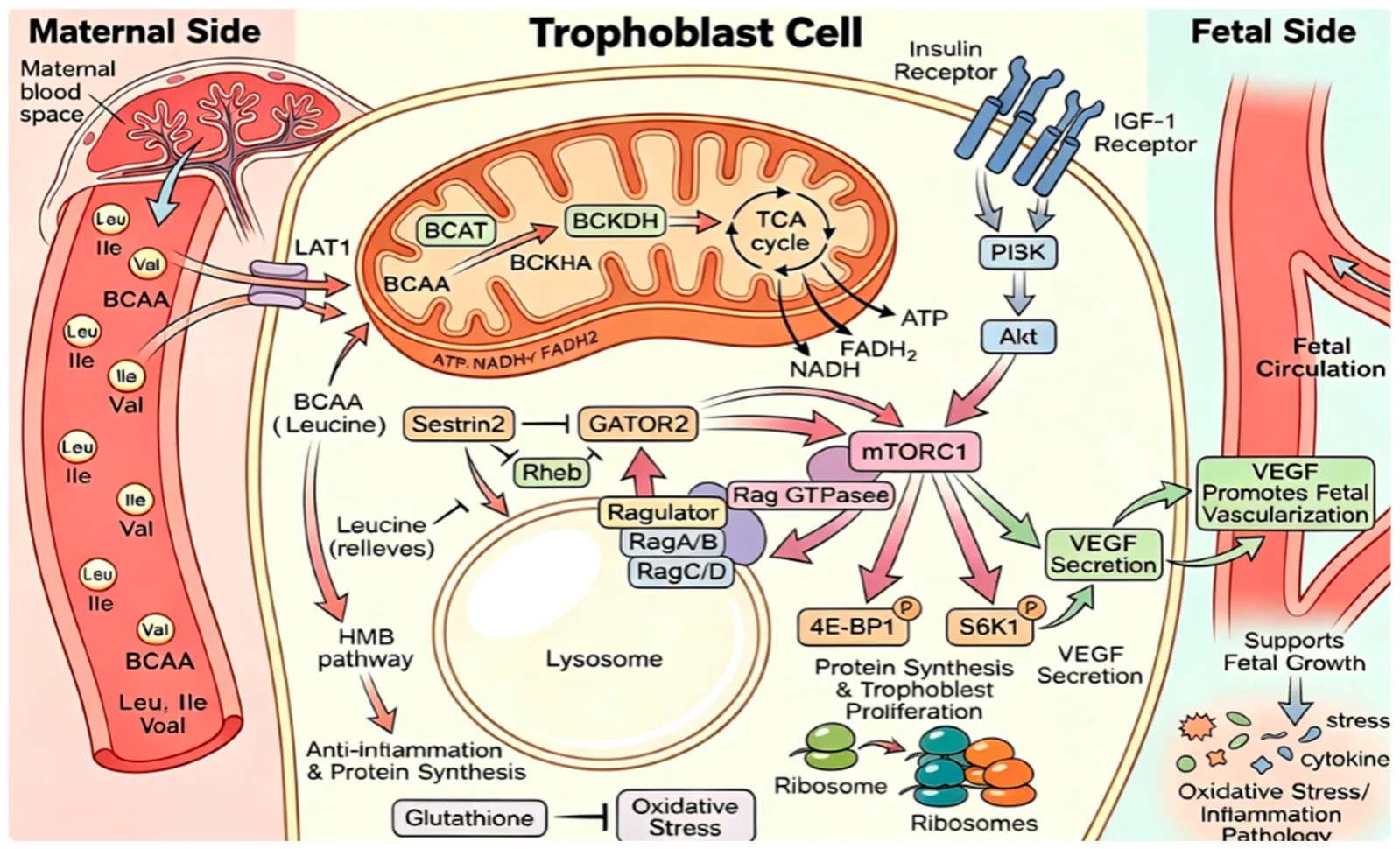
BCAA catabolism and mTORC1 signaling in placental trophoblasts. BCAAs undergo reversible transamination catalyzed by branched-chain aminotransferase (BCAT1/2) and irreversible oxidative decarboxylation by the branched-chain α-keto acid dehydrogenase (BCKDH) complex to enter the TCA cycle, generating ATP and precursors for glutathione (GSH) synthesis. Leucine activates mTORC1 via the Sestrin2–GATOR2–Rag GTPase axis, with upstream input from insulin/IGF-1 receptors through PI3K/Akt. Upon activation, mTORC1 phosphorylates 4E-BP1 (releasing eIF4E for cap-dependent translation initiation) and S6K1 (enhancing ribosomal biogenesis), promoting protein synthesis and VEGF secretion. The transcription factor EB (TFEB), regulated by mTORC1, links nutrient sensing to trophoblast syncytialization. Created in Biorender. Xinhang Li. (2026) https://BioRender.com.

The placenta carries a number of important BCAA catabolic enzymes: branched-chain aminotransferase (BCAT) and branched-chain α-keto acid dehydrogenase complex (BCKDH). BCAT carries reversible transamination of BCAAs (valine, leucine, isoleucine) into the branched-chain α-keto acids (BCKAs) and glutamate and oxidatively decarboxylates them, channeling products into downstream metabolism, where some of the maternally obtained BCAAs can be converted to relevant metabolites supporting placental function and fetal development. BCAA catabolism in the placenta also carries BCKAs that are oxidized by the TCA with adenosine triphosphate (ATP), which is responsible for high demands on placental energy for active transport of nutrients, synthesis of proteins, and cell proliferation. Metabolism involving valine, leucine, and isoleucine degradation is highly active in placental tissue, and disruption of these pathways can compromise placental function (e.g., spontaneous miscarriage in yak or preeclampsia in humans) [33,34]. Although ref. [33] reports findings from human preeclampsia—a condition without a direct porcine equivalent—the shared involvement of BCAA catabolic enzymes (BCAT2) in placental trophoblast function across species suggests conserved metabolic pathways that may inform our understanding of BCAA metabolism in the normal porcine placenta. The energy of BCAA catabolism thus contributes to the structural and functional stability of the placenta. In addition to energy production, BCAA yields glutamate, which can be further converted to glutamine or proline. Glutamine is key for nucleotide synthesis and is a nitrogen source during various metabolic reactions, and proline is important for collagen production and is important in placental extracellular matrix stability. Further, glutamate and its derivatives can serve as signaling molecules and precursors for antioxidants too; for example, glutamate can be a substrate for glutathione synthesis, a critical intracellular antioxidant that prevents placental cells from oxidative stress (a common pathological feature in placental dysfunction) [31,35]. Glutamate-derived glutathione is critical for maintaining placental redox homeostasis and maintaining trophoblast viability. Branched-chain amino acids (BCAAs) such as leucine have important roles in placental nutrient metabolism, and their catabolic enzymes can have important regulatory effects on placental function [36]. One metabolite from leucine degradation is β-hydroxy-β-methylbutyrate (HMB) that can promote protein synthesis and reduce protein breakdown and can support immune responses. HMB immunomodulatory properties in the porcine placenta may change the inflammatory environment and potentially lead to placental inflammation and fetal development. The anti-inflammatory effect of HMB may promote placental homeostasis by reducing excessive inflammatory responses that often disrupt placental function and fetal growth. In particular, inflammation is known to cause intrauterine growth restriction (IUGR) in pigs, as inflammatory cytokines may disrupt trophoblast growth and migration, both important processes for placental development and nutrient exchange [37]. In addition to HMB, BCAA catabolism generates reducing equivalents that can generate NADH and FADH2, and these cofactors are important parts of cellular redox balance and also energy metabolism. They enter the mitochondrial electron transport chain. This process regulates oxidative phosphorylation and controls the cellular oxidative state. Redox homeostasis is modulated by NADH and FADH2. These can regulate oxidative stress signaling in placental cells. Oxidative stress, arising from an imbalance between ROS production and antioxidant defenses, can compromise placental function in sows, contributing to adverse outcomes such as low birth weight and stillbirth [38]. By varying the NAD+/NADH ratio, BCAA metabolism may increase the activity of redox-sensitive enzymes and transcription factors like sirtuins. These regulate mitochondrial production and antioxidant responses. Fermented porcine placenta peptides have been shown to increase the NAD+/NADH ratio and activate sirtuin signaling pathways, which leads to mitochondrial function and senescence [39]. Thus, BCAA-derived NADH and FADH_2_ may protect the placenta by maintaining mitochondrial efficiency and enhancing antioxidant defense. In conclusion, BCAA catabolism products (HMB, NADH, FADH2, etc.) contribute to placental activity in several ways. Overall, BCAT and BCKDH facilitate BCAA metabolism of BCKAs and glutamate, integrating amino acid metabolism with energy production, antioxidant defence, and biosynthetic pathways involved in placental metabolic homeostasis. Leucine is known to be a maximal activator of the mechanistic target of rapamycin complex 1 (mTORC1), the critical regulator of cell growth and metabolism. mTORC1 activation by leucine involves Rag GTPase-mediated pathways that recruit mTORC1 to the lysosomal surface, which is key for its activation. Leucine sensing is mediated by Rag GTPases (RagA or RagB bound to RagC or RagD), which bind mTORC1 and translocate to the lysosomal membrane, where small GTPase Rheb is located. The lysosomal location is necessary for mTORC1 kinase activity, as Rheb directly stimulates mTORC1 once at the lysosome. Ragulator complex and vacuolar H+-ATPase also control Rag GTPase activity given amino acid availability [29]. Upon activation, mTORC1 phosphorylates downstream effectors, such as 4E-BP1 and S6K1 [40]. Phosphorylation of 4E-BP1 releases eIF4E, allowing the assembly of the eIF4F complex and initiation of cap-dependent translation, while S6K1 phosphorylation enhances ribosomal biogenesis and translation elongation. In placental trophoblast cells, these processes drive the proliferation and hypertrophy of the trophoblast layer, essential for placental development and function [30]. Moreover, mTORC1 signaling regulates the synthesis and secretion of placental growth factors, such as VEGF, promoting placental vascularization and increasing the surface area for nutrient exchange. At the molecular level, leucine sensing can involve the dissociation of the inhibitory Sestrin2-GATOR2 complex. When leucine binds to Sestrin2, it releases GATOR2. This can activate the Rag GTPases and help recruit mTORC1 to lysosomes [41]. Also, post-translational modifications can regulate leucine-stimulated mTORC1 activation. For example, crotonylation of calnexin (CANX) mediated by lysine acetyltransferase 7 (KAT7) is found to be a regulator. These findings illustrate the complexity of leucine sensing and mTORC1 regulation [42]. Next, leucine-induced mTORC1 activation can influence autophagy by phosphorylating ULK1. Also, the transcription factor EB (TFEB) is implicated in trophoblast syncytialization, and this links nutrient sensing to placental development [43]. In summary, leucine activates mTORC1 through Rag GTPase-dependent lysosomal recruitment, phosphorylating downstream effectors that drive protein synthesis and cell proliferation while simultaneously regulating autophagy and angiogenic factor production. The mTOR pathway in the placenta can act as a key integrator of diverse maternal nutritional and hormonal signals. These include BCAAs, insulin, IGF-1, and leptin. This pathway plays a central role in regulating fetal growth and development. BCAAs, particularly leucine, are potent activators of mTOR signaling that promote protein synthesis and placental nutrient transport capacity [44]. Hormonal signals like insulin and IGF-1 stimulate PI3K/Akt signaling upstream of mTOR and promote the expression of fetal nutrient transporters. Leptin promotes placental mTOR signaling by linking maternal energy stores to placental function. BCAAs thus modulate mTOR activity to match nutrient supply with fetal demand, enhancing mTOR signaling under nutrient-depleted conditions and potentiating hormonal signals during nutritional stress. Together, the mTOR-HIF-1α axis integrates BCAA availability with placental vascular development, nutrient transporter expression, and metabolic adaptation, supporting fetal nutrient supply matching maternal nutritional status (Figure 2).

## 4. Effects of BCAAs on Angiogenesis, Endocrine Function, and Inflammation

### 4.1. Angiogenesis and Nutrient Transport

**Figure 3:**
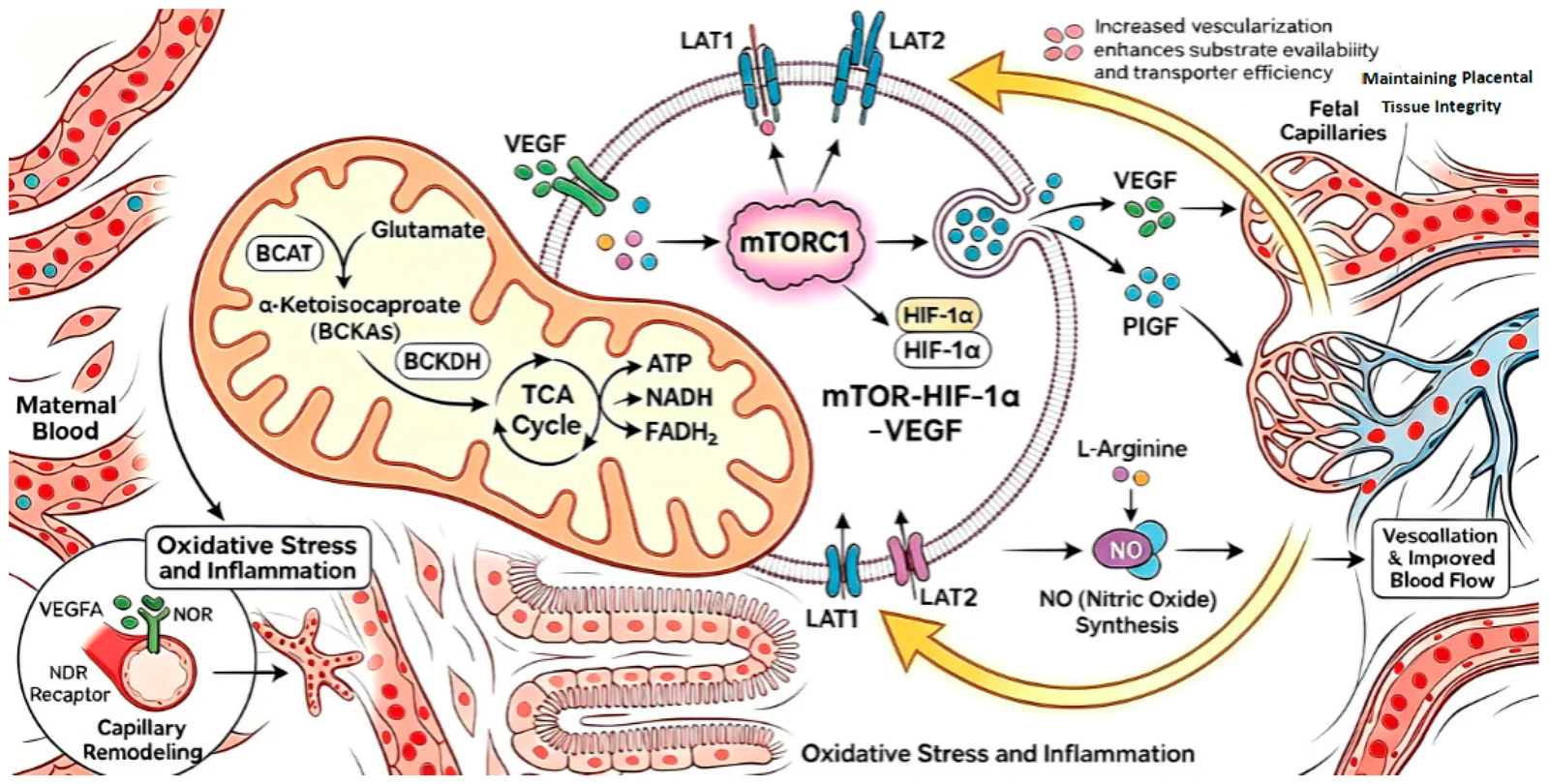
BCAA–mTOR–HIF-1α–VEGF angiogenesis feedback loop in the porcine placenta. BCAAs activate mTORC1, stabilizing HIF-1α to promote VEGF and placental growth factor (PlGF) secretion, driving angiogenesis and capillary remodeling that reduces diffusion distance between maternal and fetal circulations. L-Arginine, transported via cationic amino acid transporters (CATs/SLC7A family) and system y+L, is converted to nitric oxide (NO) by endothelial nitric oxide synthase (eNOS), mediating vasodilation and improved placental blood flow. A positive feedback loop exists whereby enhanced vascularization increases substrate availability and transporter efficiency, reinforcing BCAA-driven angiogenesis. Disruptions in this loop (e.g., preeclampsia, maternal obesity) can degrade placental blood flow and reduce BCAA supply to the fetus. Created in Biorender. Yiru Wang. (2026) https://BioRender.com.

Branched-chain amino acids (BCAAs), particularly leucine, lead to placental blood flow by activating the mTOR pathway of hypoxia-inducible factor-1α (HIF-1) stabilization. mTOR/HIF/1α activation leads to vascular endothelial growth factor (VEGF) and placental growth factor (PlGF), which are both pro-angiogenic factors that produce and remodel the placental villous capillary network, providing surface area and efficiency of maternal–fetal exchange connection, facilitating nutrient absorption, including BCAAs, to the growing fetus. VEGF and PlGF stimulate new blood vessel formation and remodeling of existing vasculature in placental vessels (VEGFs), which can be used to meet increasing fetal metabolic demands. BCAAs may also modulate vascular tone through nitric oxide (NO) synthesis, which is a powerful vasodilator facilitating placental flow and nutrient exchange efficiency. Dietary BCAA supplementation studies in pigs suggest systemic effects that may be present in placental blood flow and inflammatory responses by leucine and valine, which can indirectly contribute to angiogenesis by maintaining placental tissue [45]. Overall, BCAAs encourage placental angiogenesis through mTOR-HIF-1α-VEGF signaling, which supports vascular development in order to facilitate nutrient and oxygen transport. Porcine models suggest that angiogenic factors such as VEGFA and its receptor KDR are highly expressed on the maternal side of the placenta, with increased capillary density and surface area that facilitate nutrient exchange via a reduction in diffusion distances. The nutrient uptake in swine has been shown to improve amino acid availability, which has improved placental gene expression for angiogenesis and nutrient transportation. For example, L-arginine supplementation during early gestation upregulates genes involved in nitric oxide and polyamine synthesis, promoting angiogenesis and placental growth and increasing expression of amino acid transporters [46,47]. BCAAs can act as similar signaling molecules, and they integrate metabolic cues. These cues can help regulate placental angiogenesis and nutrient transport. BCAAs may regulate this coupling as upstream metabolic signals that coordinate vascular growth with transporter expression, ensuring synchronized nutrient delivery. Oxidative stress and inflammatory status in the placenta can affect angiogenesis and nutrient transport. BCAAs may reduce oxidative damage and help maintain vascular integrity. Antioxidants such as cysteamine reduce oxidative stress and enhance angiogenesis in the porcine placenta, and reproductive outcomes are improved [32]. As BCAAs can influence redox balance and cellular metabolism, they may support angiogenic processes and nutrient transport by maintaining placental homeostasis. In fact, the coupling of placental angiogenesis and nutrient transport can be regulated in part by BCAAs acting as upstream signals. Adequate vascularization can ensure substrate availability for nutrient transporters. At the same time, efficient transport can support vascular cell proliferation. This creates a positive feedback loop. The loop is important for placental function and fetal development. If we understand the molecular mechanisms by which BCAAs coordinate these processes, we can provide a reference for nutritional interventions to improve placental efficiency and fetal growth in swine and other mammals (Figure 3).

### 4.2. Endocrine Function and Immune Regulation

The placenta is a key endocrine system in pregnancy. It can synthesize and release hormones, such as progesterone and estrogens, and also placental lactogens, which can help maintain the pregnancy. Also, it can support fetal development. BCAAs, especially leucine, are both substrates for protein synthesis and signaling molecules that promote placental steroidogenesis through the mTOR pathway. Leucine impacts placental hormone synthesis by activating mTOR, which regulates the expression of the steroidogenic acute regulatory protein (StAR), which facilitates cholesterol flow into mitochondria, the rate-limiting step in steroid hormone synthesis, such as progesterone. Upregulating StAR expression via mTOR can increase progesterone production, which leads to the hormonal conditions required for pregnancy stability. The mTOR mechanism is known to respond to amino acid availability and regulate trophoblast proliferation, nutrient transport, and hormone production. Overall, BCAA influences placental hormones by serving as both metabolic substrates and signaling genes that activate key regulatory pathways, such as mTOR. Future studies should explore how BCAA regulation during gestation may optimize placental endocrine function and improve reproductive outcomes. Recent experiments from porcine cells suggest that amino acid availability is associated with placental steroidogenesis. In cultured porcine trophoblast cells, enhancement of branched-chain amino acids (especially leucine) can improve expression of cytochrome P450 side-chain cleavage (CYP11A1) and 3β-hydroxysteroid dehydrogenase (3β-HSD), key enzymes for progesterone and estrogen production [48]. In addition, amino acid activation of mTORC1 is found to regulate sterol regulatory element-binding protein 1 (SREBP1), which determines the lipid availability of steroid hormones and is linked to placental endocrine production. In addition to progesterone, BCAAs may affect the production of PLs that regulate maternal metabolic response during pregnancy. PPL levels of porcine placental lactogen (pPL) correlate with the placental mass and trophoblast proliferation rates, both regulated by mTORC1 activity. As BCAAs are effective mTORC1 activators, it is likely that sufficient BCAA nutrition allows pPL production, although the clinical results from swine are still poorly available and may need to be further investigated. The coupling between BCAA metabolism and placental hormone production is not limited to steroidogenesis; thyroid hormone regulation and insulin-like growth factor (IGF) axis modulation are discussed. Placental type 3 iodothyronine deiodinase (DIO3) regulates thyroid hormones at the maternal–fetal site by nutrient availability and mTOR signaling. As thyroid hormones play an important role in fetal neurodevelopment and placental angiogenesis, BCAA-mediated modulation of DIO3 activity is another way that dietary amino acids may affect fetal development [49]. Furthermore, the placental IGF axis, including IGF-1, IGF-2, and their binding proteins (IGFBPs), is responsive to amino acid supply. BCAAs may enhance local IGF-1 signaling through mTORC1-mediated upregulation of IGF-1 receptor expression, thereby amplifying growth factor signaling at the maternal–fetal interface and promoting trophoblast proliferation and differentiation [50]. Thus, BCAAs influence placental hormone production by serving as both metabolic substrates and signaling molecules that regulate steroidogenic enzyme expression through mTORC1. During pregnancy, the placenta maintains immune tolerance, protecting the semi-allogeneic fetus from maternal immune rejection, and maintaining the ability to resist infections. This delicate balance is mediated by multiple immune control mechanisms at the feto-maternal interface. BCAAs and their metabolites, particularly β-hydroxy-β-methylbutyrate (HMB), play a key role in this immune microenvironment through anti-inflammatory mechanisms. HMB can promote anti-inflammation via the inhibition of NF-κB signaling pathways [51], which can reduce excessive inflammatory reactions inside the placenta and contribute to pregnancy homeostasis, and even the porcine trophoblasts express non-classical MHC class I molecules (SLA class Ib), which are thought to play a role analogous to human HLA-G in mediating immune tolerance at the maternal–fetal interface; the absence of classical SLA class II expression on trophoblasts reduces direct activation of maternal CD4^+^ T cells and activation, which further aid immune tolerance [52]. A critical component of immune tolerance is the function of regulatory T cells (Tregs), which suppress maternal immune responses against fetal antigens. During gestation, both thymus-derived Tregs and peripherally induced Tregs contribute to the establishment of immune tolerance. Studies have demonstrated dynamic changes in paternal antigen-specific Tregs at the decidua throughout pregnancy, with neuropilin-1-positive Tregs predominating in early pregnancy and negative Tregs becoming more prominent in late pregnancy [53]. In porcine pregnancy, Tregs accumulate at the endometrium during gestation, and their expansion is influenced by the local cytokine milieu including TGF-β and IL-10 [54]. BCAAs can affect this immune balance, and they modulate Treg function, which can support expansion of regulatory T cells in the immune system, and this process is expected to help maintain the tolerance status during pregnancy. Also, ATP-binding cassette transporter member A1 (ABCA1) can downregulate TLR4 expression. It can reduce pro-inflammatory cytokine production at the feto-maternal interface [55]. In fact, BCAAs and their metabolites can regulate the placental immune microenvironment, which means they can modulate inflammatory pathways and support Treg-mediated tolerance, and they also maintain Th1/Th2 balance, which can be essential for successful pregnancy.

## 5. Effects of BCAA Deficiency or Excess on Placental Function

### 5.1. BCAA Deficiency and Intrauterine Growth Restriction

When maternal diet lacks branched-chain amino acids (BCAAs) or placental transport of these nutrients is impaired, fetal development can be disrupted. This condition may lead to intrauterine growth restriction (IUGR). The placenta serves as the interface for nutrient exchange between mother and fetus, relying heavily on the mechanistic target of rapamycin (mTOR) signaling pathway to control protein synthesis and angiogenesis, which are both essential for placental function and fetal growth. When maternal BCAA supply is not enough or placental transport mechanisms are compromised, mTOR signaling can be suppressed. This leads to reduced protein synthesis and impaired angiogenesis in the placenta [56]. This cascade results in placental insufficiency with reduced nutrient delivery to the fetus and then growth restriction [57,58]. In morphology, placentas from IUGR piglets show some abnormalities, including underdeveloped villous structures and decreased vascular density. These defects impair the placenta’s ability to support fetal demands, and it is worth noting that transcriptomic analyses have revealed downregulation of amino acid transporter genes and proteins, such as system L transporter LAT1 (Slc7a5) and system A transporters, which are critical for BCAA uptake and transplacental transfer [59]. These molecular alterations are related to reduced placental mTOR activity, further exacerbating nutrient transport deficiencies [60,61]. The reduced expression of amino acid transporters can compromise the placental amino acid flux, especially leucine, isoleucine, and valine, and these amino acids are essential for fetal protein accretion and growth signaling. Experimental models show the importance of BCAAs in placental and fetal development. For instance, trophoblast-specific overexpression of Slc7a5 in mice can enhance placental leucine transport and activate mTOR signaling, which promotes fetal growth, demonstrating the mechanistic link between BCAA transport, mTOR pathway activation, and fetal anabolic processes [62,63]. On the other hand, maternal undernutrition or toxic exposure that reduces circulating BCAA levels can lead to placental dysfunction and fetal growth retardation. Maternal cadmium exposure, for example, causes metabolic reprogramming in the maternal liver that increases amino acid uptake and metabolism, and this depletes systemic BCAA availability for placental transfer and fetal utilization, causing IUGR [64]. At the cellular level, BCAA deficiency can impair trophoblast differentiation and syncytialization, which are essential for building an effective maternal–fetal exchange surface. The mTORC1 downstream transcription factor TFEB has been identified as a key regulator of trophoblast syncytialization, with its expression positively correlating with mTOR activity and placental vascular development. Suppression of mTOR signaling due to BCAA insufficiency leads to impaired TFEB function, resulting in defective syncytiotrophoblast formation and compromised placental vascularization, thereby limiting nutrient supply to the fetus [65].

IUGR placentas also exhibit altered expression profiles of immune and inflammatory genes, with enrichment in pathways related to cytokine signaling and immune responses, which can impair placental function and nutrient transport capacity [66]. The combination of reduced amino acid transport and impaired angiogenesis, along with an altered immune milieu, can lead to placental insufficiency and contribute to fetal growth restriction. Collectively, these findings delineate a mechanistic framework wherein maternal BCAA deficiency or placental transport dysfunction suppresses mTOR signaling, reduces protein synthesis and angiogenesis, and impairs trophoblast differentiation, leading to placental insufficiency and diminished nutrient supply. This cascade is related to morphological and molecular abnormalities observed in IUGR placentas. In fact, BCAAs play a key role in fetal growth and development. Therapeutic strategies aimed at restoring placental BCAA transport and mTOR pathway activity, and they can mitigate IUGR and improve neonatal outcomes [67,68].

### 5.2. BCAA Excess and Metabolic Programming Abnormalities

Excessive branched-chain amino acids (BCAAs), particularly leucine, can disrupt the amino acid balance in the placenta and fetal circulation. This can lead to metabolic programming abnormalities with long-lasting effects. When maternal BCAA levels are high, the amino acid spectrum can become imbalanced. This ‘amino acid toxicity’ can interfere with how other essential amino acids are transported and used, and these amino acids are critical for fetal development. This imbalance can affect placental function because it changes amino acid transporter expression and activity, reducing nutrient delivery to the fetus. Studies in swine models have shown that high leucine concentrations can dysregulate protein turnover pathways in mammary epithelial cells. This happens through activation of mechanistic target of rapamycin (mTOR) signaling and suppression of proteolysis, suggesting that similar mechanisms may operate in the placenta under BCAA excess [69]. When maternal BCAA levels are elevated, fetal insulin and insulin-like growth factor-1 (IGF-1) can increase too early. These hormones can set the metabolic set point of offspring. This early hormonal surge may predispose offspring to develop postnatal obesity and metabolic syndrome, a process termed ‘metabolic programming’. Evidence from piglet models shows that BCAA supplementation or imbalance can modulate hepatic IGF-1 expression along with altered plasma metabolomic profiles, linking amino acid nutrition to growth and metabolic outcomes. Excess BCAAs, particularly leucine, can activate mTOR and inhibit autophagy in placental cells. This can lead to metabolic stress and functional disturbances. For example, exposure to high ammonia levels in pigs can increase BCAA concentrations and it upregulates mTOR signaling and downregulates AMP-activated protein kinase (AMPK), and this results in enhanced lipid synthesis and impaired β-oxidation, and it highlights the metabolic stress induced by BCAA excess [70]. Excessive BCAAs can also provoke oxidative stress and inflammatory responses in tissues. Altered cytokine profiles in piglets challenged with lipopolysaccharide and supplemented with BCAA mixtures can prove this [71]. In terms of mechanism, excess leucine competitively inhibits transport and metabolism of isoleucine and valine through shared transport systems (system L) and catabolic enzymes (BCAT and BCKDH) [72]. This leads to relative deficiencies of these two BCAAs, even when overall BCAA is in excess. This amino acid imbalance can trigger the ‘leucine paradox’. Elevated leucine concentrations, while acutely stimulating protein synthesis via mTORC1, chronically deplete circulating levels of all three BCAAs by enhancing their catabolism in peripheral tissues through shared BCAT and BCKDH enzymes. This competitive inhibition occurs at multiple levels: (1) system L transporters (LAT1/LAT2) preferentially exchange leucine for other large neutral amino acids; (2) BCAT catalyzes transamination of all three BCAAs, with leucine-derived α-ketoisocaproate (KIC) competing for the same enzyme; and (3) the BCKDH complex catabolizes all three BCKAs, with leucine-derived metabolites potentially saturating the complex. In finishing pigs, excess dietary leucine (>1.5% of diet) reduced feed intake by 10–15% and impaired growth performance through this antagonistic mechanism [73,74]. In the gestational context, excessive leucine supplementation intended to stimulate placental mTORC1 may inadvertently reduce isoleucine and valine availability for both placental and fetal metabolism, underscoring the importance of maintaining balanced BCAA ratios. At the placental level, chronic mTORC1 hyperactivation by excess BCAAs may induce endoplasmic reticulum (ER) stress through the PERK-eIF2α-ATF4 pathway, leading to impaired trophoblast function and increased apoptosis. ER stress in placental cells has been implicated in the pathogenesis of preeclampsia and gestational diabetes, conditions associated with altered amino acid metabolism [75]. Furthermore, under-dosing and excessive BCAAs can result in placental inflammation by activating the NLRP3 inflammasome and producing IL-1β and IL-6, which are pro-inflammatory conditions that affect nutrient transport and trophoblast viability. These results suggest that deficiency and excess BCAAs may be pathological states of placental functions and that precise BCAA dosing and ratio optimisation are crucial in gestational nutrition programs. From a fetal metabolic programming perspective, maternal BCAA excess during gestation can lead to epigenetic changes of fetal metabolic genes, such as DNA methylation of promoter genes involved in insulin signaling and lipid metabolism, and it is worth noting that these changes may persist. Animal studies in rodent models show that maternal high-protein diets increase circulating BCAA levels and that liver DNA methylation and gene expression in offspring are altered, and this can lead to metabolic dysfunction in later life. Direct evidence from swine is limited, but the conserved nature of these epigenetic processes suggests that similar programming effects can occur in piglets born to sows fed excessive BCAA during gestation [76]. Collectively, these findings suggest that the relationship between maternal BCAA intake and fetal outcomes follows a U-shaped curve, wherein both insufficient and excessive BCAA supply can compromise placental function and fetal development at the same time. Optimal supplementation requires precise calibration of BCAA levels and ratios throughout gestation. In summary, excessive maternal BCAA intake disrupts amino acid balance and hyperactivates mTORC1 and induces metabolic stress, which can cause long-term metabolic programming abnormalities in offspring.

## 6. Nutritional Strategies and Future Perspectives

### 6.1. Stage-Specific BCAA Supplementation

Optimizing branched-chain amino acid (BCAAs: leucine, isoleucine, and valine) during sow gestation to maximize placental development and fetal development. Placental growth and fetal nutrient requirements demand stage-specific variations in the total levels of BCAAs and ratios between leucine, isoleucine, and valine for efficient utilization and to avoid conflict. Although comprehensive sow-specific evidence remains limited, emerging data from preliminary studies have explored stage-specific BCAA supplementation paradigms: early gestation is characterized by rapid placental tissue growth and differentiation and requires a balanced amount of BCAAs to support protein accumulation and cellular metabolism. Midgestation is accompanied by continued placental expansion and fetal organogenesis, and the amount of BCAAs needs to be tuned to satisfy increasing fetal amino acid requirements. Late gestation is the period of maximum fetal growth and nutrient transfer. The number of amino acid carriers in the placenta increases, and the metabolic efficiency of the placenta is increased. Dietary BCAAs should reflect the need for fetal protein uptake and metabolism. Pig models indicate the need to adjust BCAA levels by gestational stage. For example, it has been shown that leucine is beneficial for insulin sensitivity and lipid metabolism in sows and pigs, therefore improving fetal growth as well as postnatal development. Also, balancing BCAAs is important because excess leucine can inhibit the catabolic enzymes that degrade BCAAs, which can result in deficiency in isoleucine and valine when the ratio is unbalanced, which can reduce feed intake and make growth performance worse. Ratios of approximately 2:1-2 (leucine:isoleucine:valine) can help lipid metabolism and improve meat quality in finishing pigs, with possible benefits for nutrient partitioning during gestation. BCAA levels and ratios should be adjusted with consideration of their interactions with other nutrients, including arginine, glutamine, and glucose, which can optimize placental function and fetal growth. Arginine can increase uteroplacental blood flow and help nutrient transmission. Glutamine can enhance immunity, and glucose is the main energy source for the placenta and fetus. BCAAs co-supplemented with these nutrients can help. Co-supplements of various nutrients should account for their interplay with other nutrients, e.g., arginine, glutamine and glucose, which can enhance uteroplacental blood flow and fetal development [77]. As an additional consequence, a diet including functional amino acids (nucleotides or laminarin) can improve sow reproductive performance and piglet growth due to immune responses and gut microbes that may regulate BCAA uptake and utilization. Stage-specific BCAA intake in sow diets should be taken into consideration: Early gestation (days 1–35) provides a balanced BCAA (near 2:1:2 for leucine:isoleucine/valine) for placental attachment and initial vascularization at NRC (2012) requirements. Midgestation (days 36–90) provides gradual increases in total BCAA support (10–15% above maintenance requirements) to account for growing placental mass and fetal organogenesis. Late gestation (days 91–114), when fetal growth grows very rapidly, leucine supplementation at 0.3–0.5% of diet dry matter increases placental mTORC1 activation and amino acid transport capacity and increases piglet birth weight by 8–12% [44,78]. However, excessive leucine during this period should be avoided to prevent antagonism with isoleucine and valine; maintaining the leucine:isoleucine:valine ratio at approximately 2:1:2 is recommended [74]. Arginine supplementation is at 0.4–0.8% of diet during early to mid gestation, and this is expected to improve placental vascular development through NO-mediated vasodilation, which can work together with BCAAs on angiogenesis [45]. Methionine supplementation (0.03–0.05% above requirement) can support placental SAM-dependent methylation reactions. Also, it can help VEGF-A translation via the MAT2A-mTORC1 axis [79]. Then, folic acid (15 mg/kg diet) during the periconceptional period and early gestation can support one-carbon metabolism and DNA methylation. These pathways complement BCAA-mediated signaling through their metabolic interconnections. Precision BCAA supplementation during sow gestation should be adjusted according to placental developmental stages, and attention should be paid to BCAA ratios and synergistic interactions with other functional nutrients (Table 2). A significant challenge in interpreting the reviewed literature is the absence of standardized BCAA supplementation protocols across studies. Reported doses range from 0.3% to 1.5% of diet dry matter for individual BCAAs, with supplementation periods varying from single gestational stages to the entire gestation and leucine:isoleucine:valine ratios varying considerably. Furthermore, most studies report outcomes at a single dose level, precluding systematic dose–response evaluation. Several confounding variables—including maternal parity, litter size, environmental conditions (heat stress, housing, pathogen exposure), and basal diet composition—may also influence BCAA–outcome relationships. Maternal age and parity critically govern nutrient partitioning: primiparous gilts prioritize BCAAs for maternal lean accretion, creating maternal–fetal nutrient competition, whereas hyper-multiparous sows often exhibit uterine vascular senescence, oxidative stress, and attenuated placental mTORC1 responsiveness [80,81]. Concurrently, large litter sizes in hyperprolific lines induce severe intrauterine crowding, which restricts individual placental surface area and triggers local hypoxia and ER stress, thereby downregulating LAT1/LAT2 transporter expression and dampening mTORC1 signaling across heterogeneous placental microdomains [82,83]. Furthermore, environmental stressors such as seasonal heat stress reduce uteroplacental blood flow via peripheral vasodilation, downregulating placental nutrient transporter expression and vascular remodeling [84,85], while inflammatory or sanitary challenges divert circulating BCAAs away from the conceptus toward maternal hepatic immune responses [69,76]. Beyond these confounders, the absence of standardized supplementation protocols (doses spanning 0.3–1.5% DM) and lack of multi-dose designs preclude systematic dose–response evaluations. Future in vivo studies should, therefore, implement standardized BCAA regimens and statistically adjust for parity, litter size, and environmental indices (such as the temperature-humidity index) using mixed-effects modeling.

### 6.2. Novel Additives and Functional Amino Acids

New feed additives and functional amino acids like branched-chain amino acid (BCAA) metabolites such as HMB and their analogs may assist placental function in sows by safer and more efficient modulation of key signaling processes such as mTOR and angiogenesis. BCAAs like leucine, isoleucine, and valine are key amino acids that can impact protein synthesis and cell metabolism. Recent studies have shown that mTOR signaling is key for placental nutrient transport and vascular development, which has important implications for fetal growth and survival. For example, cortisol signaling in porcine placenta activates mTORC1 signaling in a porcine placenta, thereby increasing glucose and amino acid transport by increasing transporters GLUT3 and LAT2 and improving placental function and fetal growth [86]. However, direct BCAAs may be limited by metabolic imbalance or safety considerations, and metabolites like HMB, a leucine product, or structurally similar substances may be more suitable feed components that adjust mTOR signaling and angiogenesis for safer responses. HMB has been reported in other species to promote muscle protein production and proteolysis through mTOR activation and may be mechanistically applied to modulate mTOR pathways. By controlling mTOR activity, HMB or its variants may improve placental nutrient transporter expression and vessel endothelial growth factor (VEGF)-induced angiogenesis to improve leucine and fetal supply, which is in agreement with findings that dietary intake of functional amino acids such as arginine and methionine promotes placental angiogenesis and antioxidant response by nitric oxide synthesis and WNT3A/CTNNB1 signaling, respectively. Lastly, methionine-mTORC1-S6K1-VEGF-A was shown to be a key regulator of placental artery formation by SAM-MAT2A-mTORC1 and showed the importance of amino acid metabolism for placental vascular development [87]. In addition to single amino acid metabolites, the concept of ‘functional amino acid’ combinations is gaining traction for their synergistic effects in mitigating maternal stressors, such as heat stress and infections, which negatively impact placental function. Oxidative stress and inflammation are common challenges during gestation that impair placental angiogenesis and nutrient transport, leading to intrauterine growth restriction (IUGR) and low birth weight piglets.

### 6.3. Future Research Directions

Developing in vitro models that can mimic the complex cell types and functions of the porcine placenta is important for studying branched-chain amino acids (BCAAs) in nutrient allocation. Recently, swine trophoblast organoids (sTOs) derived from full-term porcine placentas have been shown to retain key transcriptional features and self-organize into distinct trophoblast populations that recapitulate in vivo cellular diversity [88]. When combined with spatial transcriptomics data from mid-gestation placentas, these organoids can be used as a platform to study cell type-specific roles and molecular pathways in placental development. Using such organoid systems alongside established trophoblast cell lines can help control and screen gene functions in high-throughput. Gene editing technologies, such as CRISPR/Cas9, can be used to knock out or modify genes encoding BCAA receptors (e.g., LAT1, LAT2) and amino acid transporters (e.g., SLC family members) in these models. This can reveal their contributions to BCAA uptake and signaling, and downstream effects on placental growth. This approach can avoid limitations of in vivo studies, where cellular complexity and systemic factors make it hard to get clear mechanistic insights. Also, gene-edited placental models can be used to study how BCAA transport influences trophoblast proliferation and migration, as well as angiogenesis, which are important for placental morphogenesis and function [89]. Combining gene editing with transcriptomic and proteomic profiling in these models can help identify signaling pathways regulated by BCAAs, such as mTOR and STAT3, which have been found to be related to placental and fetal muscle development. In the end, these in vitro systems thus enable detailed investigation of BCAA-related molecular mechanisms in the placenta. This can help identify therapeutic targets to optimize fetal growth and litter uniformity. A full understanding of how BCAAs regulate placental physiology requires multi-omics analysis that can capture gene expression and protein abundance, as well as metabolic fluxes in the placenta. Transcriptomic studies have shown that placental development and function are regulated by dynamic changes in gene expression, including genes for nutrient transport, angiogenesis, and cellular proliferation. However, mRNA levels do not always correlate with protein expression or metabolic activity, necessitating proteomic and metabolomic profiling. In summary, combining in vitro models and multi-omics technologies, together with long-term animal studies, is essential for translating mechanistic insights into practical nutritional strategies that improve placental BCAA metabolism and reproductive outcomes. Several limitations of the current evidence base must be acknowledged. Much of the mechanistic evidence derives from rodent models and in vitro systems; direct translation to the sow placenta requires caution given the epitheliochorial vs. hemochorial architectural differences. The number of in vivo sow studies remains limited, and many reported effects (e.g., 8–12% increase in birth weight) lack formal power analysis, raising concerns about statistical robustness. This narrative review does not include a formal meta-analysis; the heterogeneity in study designs, doses, and outcome measures precludes meaningful statistical pooling. We encourage future researchers to report effect sizes with confidence intervals and to conduct power analyses a priori. Furthermore, the quality of the reviewed studies has not been systematically assessed. Future systematic reviews should incorporate standardized quality assessment tools (e.g., the GRADE framework or the Newcastle–Ottawa Scale adapted for animal studies) to evaluate the strength and reliability of the evidence base.

## 7. Conclusions

We consider branched-chain amino acids (BCAAs), especially leucine, as important regulators for placental nutrient allocation in the sows. They function not merely as nutritional substrates. When mTORC1 signaling is activated, BCAAs can coordinate placental growth and angiogenesis while also regulating amino acid transport to support fetal development. However, both deficiency and excess of BCAAs can damage placental function through different mechanisms: deficiency can reduce mTORC1 activity, and this compromises nutrient transport capacity, while excess disturbs amino acid balance and can cause metabolic programming abnormalities. The integration of BCAA metabolism with hormonal signaling and immune regulation, together with vascular development, shows that placental nutrient sensing is complex. Future research can focus on stage-specific BCAA requirement models and multi-omics approaches to identify cell type-specific responses. We should study precision nutrition strategies. The interactions among BCAAs and other functional amino acids warrant particular attention. A comprehensive understanding of BCAA-mediated placental nutrient allocation will provide actionable insights for improving swine reproduction and piglet survival. Future research should prioritize: (1) standardized, dose-controlled BCAA intervention trials in defined sow populations with adequate statistical power; (2) multi-omics approaches (transcriptomics, proteomics, metabolomics) to identify cell type-specific responses using advanced models, such as trophoblast organoids; (3) investigation of BCAA interactions with other functional amino acids (arginine, methionine, glutamine) for integrated supplementation strategies; (4) systematic reviews with meta-analysis as the evidence base matures, with reporting of effect sizes and confidence intervals; and (5) translation of mechanistic insights into precision nutrition strategies that account for gestational stage, breed, and environmental context.

## Figures and Tables

**Table 1:** Key BCAA transporters and signaling components in the porcine placenta.

Component	Gene/Protein	Function in Placenta	References
LAT1	SLC7A5	Na^+^-independent exchange of large neutral amino acids (Leu, Ile, Val) at apical trophoblast membrane	[9,11]
LAT2	SLC7A8	Bidirectional amino acid exchange with broader substrate specificity at basal membrane	[13,28]
System B^0+^	ATB^0+^	Na^+^-dependent transport of neutral and cationic amino acids	[14]
System y+L	SLC7A6/7A7	Cationic and neutral amino acid exchange; vectorial BCAA transport	[28]
mTORC1	MTOR/Raptor	Central signaling hub integrating BCAA sensing with placental growth and transporter expression	[29,30]
HIF-1α	HIF1A	Downstream effector of mTORC1; mediates angiogenic responses	[29]
BCAT	BCAT1/2	Branched-chain aminotransferase; reversible transamination of BCAAs	[15,31]
BCKDH	BCKDHA/B	Irreversible decarboxylation of BCKAs	[32]

**Table 2:** Nutritional intervention strategies targeting BCAA-mediated placental function in swine.

Strategy	Stage	Mechanism	Outcome	References
Leucine (0.3–0.5% DM)	Late gestation	Activates mTORC1; upregulates transporters vi PI3K/Akt	↑ Birth weight 8–12%	[44,78]
BCAA ratio 2:1:2	The whole process	Prevents Leu antagonism with Ile/Val	↑ Feed intake; ↓ imbalance	[74]
Arginine (0.4–0.8%)	Early–mid gestation	NO-mediated vasodilation; polyamine synthesis	↑ Vascular density	[45,47]
Methionine (+0.03–0.05%)	Gestation	SAM-dependent methylation; VEGF-A via MAT2A-mTORC1	↑ Angiogenesis	[79]
HMB	Late gestation	Leucine metabolite; protein synthesis, anti-inflammatory	↑ Protein accretion	[37]
Folic acid (15 mg/kg)	Periconceptional	One-carbon metabolism; DNA methylation	↑ Placental development	[35]
3-HIB (0.05–0.1%)	Late gestation	Valine catabolite; promotes placental vascular development	↓ Within-litter birth weight variation	[24]
Cysteamine (100–200 mg/kg)	Gestation	Antioxidant; alleviates oxidative stress; enhances angiogenesis	↑ Placental vascular density; ↓ oxidative damage	[22]
Soy isoflavones (40 mg/kg)	Gestation	Antioxidant; enhances placental antioxidant status	↑ Antioxidant capacity; improved piglet intestinal health	[23]

Doses and ratios shown are as reported in individual studies. Systematic dose–response evaluation in sows has not been performed. Values should be interpreted with caution. ↑ Indicates an increase or improvement ↓ Indicates a decrease or weakening.

## Data Availability

No new data were created or analyzed in this study. Data sharing is not applicable to this article.

## Abbreviations

3-HIB, 3-Hydroxyisobutyric Acid; Akt, Protein Kinase B; BCAAs, Branched-Chain Amino Acids; BCAT, Branched-Chain Aminotransferase; BCKAs, Branched-Chain α-Keto Acids; BCKDH, Branched-Chain α-Keto Acid Dehydrogenase; HIF-1α, Hypoxia-Inducible Factor 1α; HMB, β-Hydroxy-β-Methylbutyrate; IUGR, Intrauterine Growth Restriction; LBW, Low Birth Weight; LAT1, L-Type Amino Acid Transporter 1; LAT2, L-Type Amino Acid Transporter 2; mTORC1, Mechanistic Target of Rapamycin Complex 1; NO, Nitric Oxide; PI3K, Phosphoinositide 3-Kinase; SNAT, Sodium-Coupled Neutral Amino Acid Transporter; VEGF, Vascular Endothelial Growth Factor; StAR, Steroidogenic Acute Regulatory Protein; NF-κB, Nuclear Factor Kappa Light-Chain Enhancer of Activated B Cells; TCA, Tricarboxylic Acid Cycle; SNAT1, Sodium-Coupled Neutral Amino Acid Transporter 1; GLUT1, Glucose Transporter 1.
